# Dyslipidemia Among Patients With Type 1 Diabetes and Its Associated Factors in Saudi Arabia: An Analytical Cross-Sectional Study

**DOI:** 10.7759/cureus.21923

**Published:** 2022-02-05

**Authors:** Abdullah A Alrasheed

**Affiliations:** 1 Department of Family and Community Medicine, King Saud University, Riyadh, SAU

**Keywords:** saudi arabia, lipid profile, prevalence study, type 1 diabetes mellitus (t1d), dyslipidemia

## Abstract

Objectives

The present study was conducted to determine the prevalence of dyslipidemia among patients with type 1 diabetes mellitus (T1DM) and its associated factors in Saudi Arabia.

Methods

An analytical cross-sectional study was conducted to examine patients with T1DM at a tertiary care hospital in Riyadh, Saudi Arabia. The hospital’s electronic medical records (EMRs) and a telephone survey were used to collect data after obtaining institutional approval and informed consent from every patient. The inclusion criteria included age >18 years, T1DM, and follow-up at the tertiary care hospital. The data collected included age, gender, hypertension, glycosylated hemoglobin (HbA1c), body mass index (BMI), diabetes duration, total cholesterol, triglycerides (TG), low-density lipoprotein (LDL), and high-density lipoprotein (HDL).

Results

A total of 234 patients with T1DM were included in the study, which comprised 44.4% men and 55.6% women with an average age of 30.9 ± 9.7 years. Total cholesterol, TG, LDL-C, and HDL-C were 4.6 ± 1.04 mmol/L, 1.1 ± 0.60 mmol/L, 2.6 ± 0.89 mmol/L, and 1.5 ± 0.43 mmol/L, respectively. About 18.4% were hypertensive, and about 51.9% and 50% men and women had dyslipidemia, respectively. Males and HbA1c ≥ 7% showed significant associations with dyslipidemia.

Conclusion

Dyslipidemia is prevalent in patients with T1DM in Saudi Arabia, which warrants the use of solid preventive strategies to limit the risk of cardiovascular disease.

## Introduction

Dyslipidemia is an important metabolic disorder that involves persistent abnormally high concentrations of plasma cholesterol and triglycerides (TG) [1]. Dyslipidemia is defined as total cholesterol ≥ 5.17 mmol/L (≥200 mg/dL), high low-density lipoprotein-cholesterol (LDL-C) ≥ 3.36 mmol/L (≥130 mg/dL), low high-density lipoprotein-cholesterol (HDL-C) < 1.03 mmol/L (<40 mg/dL) for men and < 1.3 mmol/L (<50 mg/dL) for women, and elevated TG > 1.7 mmol/L (>150 mg/dL), or a combination thereof [2]. Dyslipidemia is one of the major but modifiable risk factors for cardiovascular diseases (CVDs) such as myocardial infarction (MI) and stroke. It has been reported that one-third of cases of MI are attributable to high cholesterol [3]. Globally, LDL-C increases both mortality and morbidity, with a burden of 4.32 million deaths and 94.92 million disability-adjusted life years (DALYS) [4].

The prevalence of dyslipidemia is high in patients with T1DM [5], and T1DM increases the risk of CVD by up to 4-10 times [6]. Patients with T1DM show several qualitative and functional atherogenic lipoprotein abnormalities [7] and the prevalence of dyslipidemia among them has been reported to be 72.5%, putting them at increased risk of vascular complications [8].

In addition, it has been reported that patients with T1DM show high LDL-C and low HDL-C [6]. Patients with T1DM are at the risk of the earlier development of atherosclerosis, leading to increased morbidity and mortality [9]. Thus, if T1DM and dyslipidemia are both present, the risk of CVD is even higher. High concentrations of TG, total cholesterol (TC), and low HDL-C have positive associations with poor glycemic control, pushing the risk of diabetic complications even further [9].

The prevalence of dyslipidemia is on the rise worldwide due to Westernized diets, urbanization, poor physical activity, and obesity [10]. A sedentary lifestyle is associated with obesity, elevated TGs, reduced HDL-C, hypertension, and hyperglycemia, resulting in an increased risk of CVD and subsequent mortality and morbidity [10]. LDL-C is the eighth leading risk factor of death worldwide [11], and diabetes is the seventh leading cause [12]. Therefore, evaluation of dyslipidemia, especially among patients with diabetes, is important to enforce preventive strategies, especially among populations at risk for CVD.

In Saudi Arabia, dyslipidemia has been reported in 66.5% of patients with T2DM [13]. However, no literature is available on the prevalence of dyslipidemia in patients with T1DM. Unfortunately, Saudi Arabia stands fifth in the global incidence of T1DM [14]. Thus, Saudi populations with dyslipidemia and diabetes are at high risk of developing cardiovascular events. Therefore, this study was conducted to determine the prevalence of dyslipidemia and its associated factors in Saudi patients with T1DM.

## Materials and methods

The relevant data used in this study were extracted from a previous study [15]. An analytical cross-sectional study was conducted to examine patients with T1DM at a tertiary care hospital in Riyadh, Saudi Arabia. After obtaining consent from the Institutional Review Board (IRB: E-20-5530), a list of 900 patients registered from January 2018 to December 2020 in the hospital electronic medical records (EMRs) was accessed, and a telephone survey was conducted to collect data. Informed consent was obtained from every patient before conducting the telephone survey. The inclusion criteria included age >18 years, T1DM, and follow-up at the tertiary care hospital. The exclusion criteria included patients with incomplete data in EMRs and those who had fewer than two visits to the hospital.

A structured interview-based questionnaire was distributed among the patients, and 234 patients were selected for analysis based on the inclusion criteria. The data collected included age, gender, hypertension, glycosylated hemoglobin (HbA1c), basal mass index (BMI), diabetes duration, TC, TG, LDL-C, and HDL-C. Dyslipidemia was defined as total cholesterol ≥ 5.17 mmol/L (≥200 mg/dL), high LDL-C ≥ 3.36 mmol/L (≥130 mg/dL), low HDL-C < 1.03 mmol/L (<40 mg/dL) for men, < 1.3 mmol/L (<50 mg/dL) for women, and elevated TG > 1.7 mmol/L (>150 mg/dL), or a combination thereof [2].

The 26th version of the statistical package of social sciences (SPSS Inc., Chicago, IL, USA) was used to analyze the collected data. Descriptive statistics were used to represent the clinical and background data of the patients. The significance of clinical and background data was assessed via an independent-sample t-test and chi-squared test of independence.

## Results

The study involved 234 patients with T1DM, including 104 (44.4%) men and 130 (55.6%) women. The mean age of the patients was 30.9 ± 9.7 years, and the mean HbA1c and mean BMI were 8.8 ± 2.0 and 26.4 ± 6.30, respectively. The mean duration of T1DM from the point of diagnosis was 13.7 ± 8.07 years. TC, TG, and LDL-C, HDL-C were 4.6 ± 1.04 mmol/L, 1.1 ± 0.60 mmol/L, 2.6 ± 0.89 mmol/L, and 1.5 ± 0.43 mmol/L, respectively. About 18.4% were hypertensive. Clinical and background characteristics of the patients are given in Table 1.

**Table 1:** Clinical and background characteristics of the participants (n= 234)

Characteristics	N (%)	M ± SD
Sex		
Male	104 (44.4)	
Female	130 (55.6)	
Age		30.9±9.7
A1C mean		8.8±2.0
BMI mean		26.4±6.30
Diabetes duration		13.7±8.07
Total cholesterol		4.6±1.04
TG		1.1±0.60
LDL		2.6±0.89
HDL		1.5±0.43
Hypertensive		
Yes	43 (18.4)	
NO	191 (81.6)	

The prevalence of dyslipidemia was 51.9% in men and 50% in women (Table 2). The prevalence of LDL-C dyslipidemia was significantly higher in men than in women (P-value = 0.024, Table 2).

**Table 2:** Prevalence of dyslipidemia their mean values stratified by gender (n = 234)

		Prevalence				Mean +-SD			
	Categories	Male	Female	P-value	Total	Male	Female	P-value	Total
Dyslipidemia	Yes	54 (51.9)	65 (50)	0.7936	119 (50.9)	NA	NA	NA	NA
	No	50 (48.1)	65 (50)		115 (49.1)				
Total cholesterol	<200 mg/dL	71 (68.3)	94 (72.3)	0.5644	165 (70.5)	180.9±39.5	180.8±41.5	0.9861	180.9±40.6
	≥ 200 mg/dL	33 (31.7)	36 (27.7)		69 (29.5)				
Triglyceride	<150 mg/dL	89 (85.6)	111 (85.4)	.966913	200 (85.5)	100.9±55.9	93.9±51.3	0.3200	97.0±53.4
	≥150 mg/dL	15 (14.40)	19 (14.6)		34 (14.5)				
LDL-C	<130 mg/dL	79 (76)	114 (87.7)	0.0242	193 (82.5)	107.4±34.0	98.7±34.7	0.0557	102.6±34.6
	≥ 130 mg/dL	25 (24)	16 (12.3)		41(17.5)				
HDL-C (M/W)	<40/50 mg/dL	24 (23.1)	28 (21.5)	.778492	52 (22.2)	53.2±13.7	63.4±17.8	0.0001	58.9±16.9
	≥ 40/50 mg/dL	80 (76.9)	102 (78.5)		182 (77.8)				

There was no significant difference between men and women in terms of overall dyslipidemia (P-value = 0.79), total cholesterol (P-value = 0.56), and TG (P-value = 0.96). Males had significant dyslipidemia of LDL-C (≥130 mmol/L, P-value = 0.018). The patients with HbA1c of ≥7% had significant dyslipidemia of TC (≥200 mg/dL, P-value = 0.023). However, age (P-value = 0.097), gender (P-value = 0.76), HbA1C (P-value = 0.21), BMI (P-value = 0.24), diabetes duration (P-value = 0.074), and hypertension (P-value = 0.11) showed no statistically significant association with overall dyslipidemia. Table 3 represents the factors affecting dyslipidemia and lipid components among patients with T1DM, and Table 4 shows the co-occurrence of four dyslipidemias in terms of gender.

**Table 3:** Factors affecting the prevalence of dyslipidemia and its lipid components (n = 234)

Variables Categories	Category	Dyslipidemia (n (%))	Elevated TC (≥200 mg/dL) (n (%))	Elevated TG (≥150 mg/dL) (n (%))	High LDL-C (≥130 mg/dL) (n (%))	Low HDL-C (M/W ≥ 40/50 mg/dL) (n (%))
Age	<25	34 (28.6)	22 (31.9)	9 (26.5)	13 (31.7)	16 (30.8)
	≥25	85 (71.4)	47 (68.1)	25 (73.5)	28 (68.3)	36 (69.2)
	P-value	.097327	.361036	.19625	.498456	.3449
Gender	Male	54 (45.4)	33 (47.8)	15 (44.1)	25 (61)	24 (46.2)
	Female	65 (54.6)	36 (52.2)	19 (55.9)	16 (39)	28 (53.8)
	P-value	.769984	.500818	.966913	.018997	.778492
A1C	< 7%	13 (10.9)	4 (5.8)	3 (8.8)	3 (7.3)	8 (15.4)
	≥ 7%	106 (89.1)	65 (94.2)	31 (91.2)	38 (92.7)	44 (84.6)
	P-value	.212822	.023318	.373136	.191989	.684153
A1C	< 8%	49 (41.2)	25 (36.2)	14 (41.2)	15 (36.6)	23 (44.2)
	≥ 8%	70 (58.8)	44 (63.8)	20 (58.8)	26 (63.4)	29 (55.8)
	P-value	.44887	.142155	.758881	.319294	.91582
BMI	< 30	91 (76.5)	56 (81.2)	24 (70.6)	32 (78)	37 (71.2)
	≥ 30	28 (23.5)	13 (18.8)	10 (29.4)	9 (22)	15 (28.8)
	P-value	.245038	.682052	.164534	.801695	.091516
BMI	< 24.9	52 (43.7)	31 (44.9)	14 (41.2)	18 (43.9)	23 (44.2)
	25 – 29.9	37 (31.1)	24 (34.8)	10 (29.4)	13 (31.7)	13 (25)
	30 – 34.9	19 (16)	10 (14.5)	8 (23.5)	7 (17.1)	9 (17.3)
	≥ 35	11 (9.2)	4 (5.8)	2 (5.9)	3 (7.3)	7 (13.5)
	P-value	.341513	.413379	.248749	.766612	.396512
Diabetes duration	< 5 years	26 (21.8)	17 (24.6)	11 (32.4)	11 (26.8)	11 (21.2)
	5 - <10 years	18 (15.1)	9 (13)	4 (11.8)	6 (14.6)	9 (17.3)
	≥ 10 years	75 (63)	43 (62.3)	19 (55.9)	24 (58.5)	32 (61.5)
	P-value	.074822	.105242	.071906	.293708	.747326
Hypertension	Yes	27 (22.7)	12 (17.4)	10 (29.4)	8 (19.5)	14 (26.9)
	No	92 (77.3)	57 (82.6)	24 (70.6)	33 (80.5)	38 (73.1)
	P-value	.114471	.801405	.072303	.836143	.071155

 

**Table 4:** Co-occurrence of the four lipid abnormalities stratified by gender (n = 234) Abbreviations: LDL-C: Low-Density Lipoprotein Cholesterol, TC: Total Cholesterol, TG: Triglyceride, HDL-C: High-Density Lipoprotein Cholesterol

Lipid abnormalities	Male	Female	Total
Negative	50 (43.5)	65 (56.5)	115
TC+TG+LDL-C	6 (60)	4 (40)	10
TC+LDL-C	25 (62.5)	15 (37.5)	40
LDL-C	25 (61)	16 (39)	41
TG+LDL-C	6 (60)	4 (40)	10
TG	15 (44.1)	19 (55.9)	34
TG+HDL-C	15 (44.1)	19 (55.9)	34
HDL-C	24 (77.4)	7 (22.6)	31
LDL+HDL-C	25 (61)	16 (39)	41
TC+TG+LDL-C+HDL-C	6 (60)	4 (40)	10
TG+LDL-C+HDL-C	6 (60)	4 (40)	10
TC+TG	7 (43.8)	9 (56.3)	16
TC+TG+HDL-C	3 (60)	2 (40)	5
TC	33 (47.8)	36 (52.2)	69
TC+LDL-C+HDL-C	10 (90.9)	1 (9.1)	11
TC+HDL-C	6 (66.7)	3 (33.3)	9

## Discussion

The present cross-sectional study was conducted to determine the prevalence of dyslipidemia among patients with T1DM and its associated factors in a Saudi population. The study revealed that dyslipidemia is prevalent among the population of Saudi Arabia suffering from T1DM, with a marked risk of CVD such as MI and stroke. Male patients had significant dyslipidemia of LDL-C, and patients with HbA1c of ≥7% had significant dyslipidemia of TC.

Type 1 diabetes is an autoimmune disease with a peak incidence in children of age 10-14 years and accounts for 5% of all diabetes cases globally [16]. Dyslipidemia is frequent comorbidity found in diabetic patients with a prevalence of 72.5% or even more [8]. Abed et al. [17] conducted a cross-sectional study and reported a prevalence of dyslipidemia of 64% among 129 young adults with T1DM. Similarly, Mona et al. [18] studied 60 children and adolescents and reported rates of 65% and 28.2% of dyslipidemia in diabetic patients and controls, respectively, where high LDL-C and low HDL-C were the most common forms of dyslipidemia (P < 0.001).

T1DM is a soaring epidemic in Saudi Arabia, affecting 35,000 children. The country is eighth in the world in terms of incidence rate at 33.5 cases per 100,000 individuals [19]. Unfortunately, Alzahrani et al. [20] have reported a rate of 33% of dyslipidemia among Saudi individuals. On the other hand, Alzahib and Altemani [13] have reported dyslipidemia in 66.5% of patients with T2DM. Thus, dyslipidemia is prevalent in Saudi populations with either T1DM or T2DM, although dyslipidemia is relatively less common in patients with T1DM than in those with T2DM. The reason behind this slight difference may be attributed to age since the incidence of dyslipidemia increases with advancing age [21]. The incidence of T1DM peaks at 10-14 years, while the incidence of T2DM peaks at around 55 years of age [22].

In the present study, although overall dyslipidemia was more frequent in men than in women, this difference was not statistically significant. In addition, LDL-C was significantly higher in men than in women. LDL-C is termed as “bad cholesterol,” indicating that Saudi men are at higher risk of CVD than women. On the other hand, Homma et al. [8] conducted a retrospective cross-sectional study in Brazil including 239 young patients with T1DM. They reported overall dyslipidemia rates of 81.7% and 61.8% in females and males (P = 0.01), respectively. This difference may be attributed to the retrospective design of the study and the unknown status of statin therapy. Similarly, Bulut et al. [23] observed more females with overall dyslipidemia as compared to men.

Another important finding in the present study was that patients with HbA1c of ≥7% had significant dyslipidemia of TC. In support of this result, Abed et al. [17] also reported that higher mean HbA1c was significantly associated with dyslipidemia (P < 0.043). Therefore, men with T1DM and HbA1c of 7% or higher are at high risk of CVD. In this context, timely and strict preventive measures may reduce the risk of adverse cardiovascular events.

The present study did not show any significant association of dyslipidemia with age, BMI, diabetes duration, and the presence or absence of hypertension. In support of the present study, Mona et al. [18] reported that age (P = 0.47) and duration of diabetes (P = 0.29) showed no significant association with dyslipidemia. However, they reported a significant association of BMI (P = 0.024) with dyslipidemia. In contrast, Marcovecchio et al. [24] studied the prevalence of dyslipidemia in 895 T1DM patients and reported significant associations with age (P < 0.001), BMI (P < 0.05), duration of diabetes (P < 0.001), and HbA1c (P < 0.001). Similarly, Soliman and Ibrahim [25] retrospectively studied 806 children and adolescents with T1DM and reported that poor glycemic control was significantly associated with higher components of dyslipidemia (TG, TC, LDL-C), longer diabetes duration, and older age. These differences in the association of dyslipidemia may be explained by the missing data on statin therapy in the present study.

An advantage of this study is that it has reported the prevalence of dyslipidemia in T1DM patients for the first time in Saudi Arabia to the best of our knowledge. The present study has limitations, such as a single-centered design and not including the status of statin therapy of patients.

## Conclusions

The prevalence of dyslipidemia is high in Saudi Arabia, indicating a high risk for CVD. Therefore, further studies on the relation of dyslipidemia with T1DM and strict preventive strategies against dyslipidemia are required. Such efforts could reduce the combined risk of dyslipidemia and diabetes in the Saudi population.
